# The noncarbonic anhydrase inhibiting acetazolamide analog *N*-methylacetazolamide reduces the hypercapnic, but not hypoxic, ventilatory response

**DOI:** 10.14814/phy2.12484

**Published:** 2015-08-19

**Authors:** Luc J Teppema, Erik R Swenson

**Affiliations:** 1 Department of Anesthesiology, Leiden University Medical CentreLeiden, The Netherlands; 2 Pulmonary and Critical Care Medicine, VA Puget Sound Health Care System, University of WashingtonSeattle, Washington, USA

**Keywords:** Carbon dioxide, carbonic anhydrase, hypoxia, n-methyl acetazolamide, ventilation

## Abstract

Previous studies have shown that the carbonic anhydrase (CA) inhibitors acetazolamide (AZ) and methazolamide (MZ) have inhibiting actions on breathing. Classically these have been attributed to CA inhibition, but other effects unrelated to CA inhibition have been identified in other tissues. To explore this possibility in the control of ventilation by the central nervous system, we investigated whether an AZ-analog without CA inhibiting properties, by virtue of a single methylation on the sulfonamide moiety, *N*-methylacetazolamide (NMA), would still display similar actions to acetazolamide and methazolamide. NMA (20 mg kg^−1^) was given intravenously to anesthetized cats and we measured the responses to steady-state isocapnic hypoxia and stepwise changes in end-tidal pco_2_ before and after infusion of this AZ analog using the technique of end-tidal forcing. NMA caused a large decrease in the apneic threshold and CO_2_ sensitivity very similar to those previously observed with AZ and MZ, suggesting that these effects are mediated independently of CA inhibition. In contrast to acetazolamide, but similar to methazolamide, NMA did not affect the steady-state isocapnic hypoxic response. In conclusion, our data reveal complex effects of sulfonamides with very similar structure to AZ that reveal both CA-dependent and CA-independent effects, which need to be considered when using AZ as a probe for the role of CA in the control of ventilation.

## Introduction

The sulfonamide carbonic anhydrase (CA) inhibitor acetazolamide (AZ) has complex effects on the control of breathing (Swenson 1998; Swenson and Teppema 2007). When infused intravenously, it exerts specific actions on the peripheral chemoreceptors of the carotid bodies and respiratory muscles that partly account for a reduction in the hypoxic ventilatory response (HVR), the loss of the O_2_–CO_2_ interaction at the peripheral chemoreceptors, and a decrease in ventilatory CO_2_ sensitivity (Kiwull-Schone et al. 2001, 2006; Teppema and Dahan 2004; Teppema et al. 2006b). Methazolamide (MZ), a more lipophilic inhibitor with equal affinity for the various CA isoforms (Maren 1956), surprisingly does not share the same effects of AZ on respiratory muscles and the hypoxic response, suggesting alternative mechanisms other than CA inhibition by which AZ exerts these inhibitory actions on ventilatory control (Teppema et al. 2006a; Kiwull-Schone et al. 2009).

Actions of AZ independent of CA inhibition have been reported for resistance vessels in the human forearm (Pickkers et al. 2001), in porcine retinal arterioles (Torring et al. 2009), in rat skeletal muscle cells (Tricarico et al. 2000), hypoxic pulmonary vasoconstriction (HPV) in the dog and human (Hohne et al. 2007; Teppema et al. 2007; Ke et al. 2013; Pickerodt et al. 2014), and rat pulmonary arterial smooth muscle cells (PASMC) (Shimoda et al. 2007). In unanesthetized dogs (Hohne et al. 2007; Pickerodt et al. 2014) and rat PASMC (Shimoda et al. 2007), AZ inhibits HPV and hypoxia-mediated elevations in cytosolic calcium, respectively, while two other potent CA inhibitors, benzolamide and ethoxzolamide, structurally dissimilar in their heterocyclic ring structures are ineffective. In these same models, the effect of the non-CA inhibiting AZ analog, *N*-methylacetazolamide (NMA) was also tested. With substitution of a methyl group for one of the hydrogen ions in the sulfonamide moiety of acetazolamide, the molecule loses its CA inhibiting property (Maren 1956). Similar to acetazolamide, NMA inhibits the rise in [Ca^2+^_i_] induced by hypoxia in PASMCs (Shimoda et al. 2007) as well as inhibiting HPV in the dog (Pickerodt et al. 2014). Thus, NMA, an acetazolamide analog without CA inhibiting properties, was useful in uncovering a yet unknown mechanism by which AZ reduces the hypoxic response of PASMCs and pulmonary vasculature in vivo.

**Figure 1:**
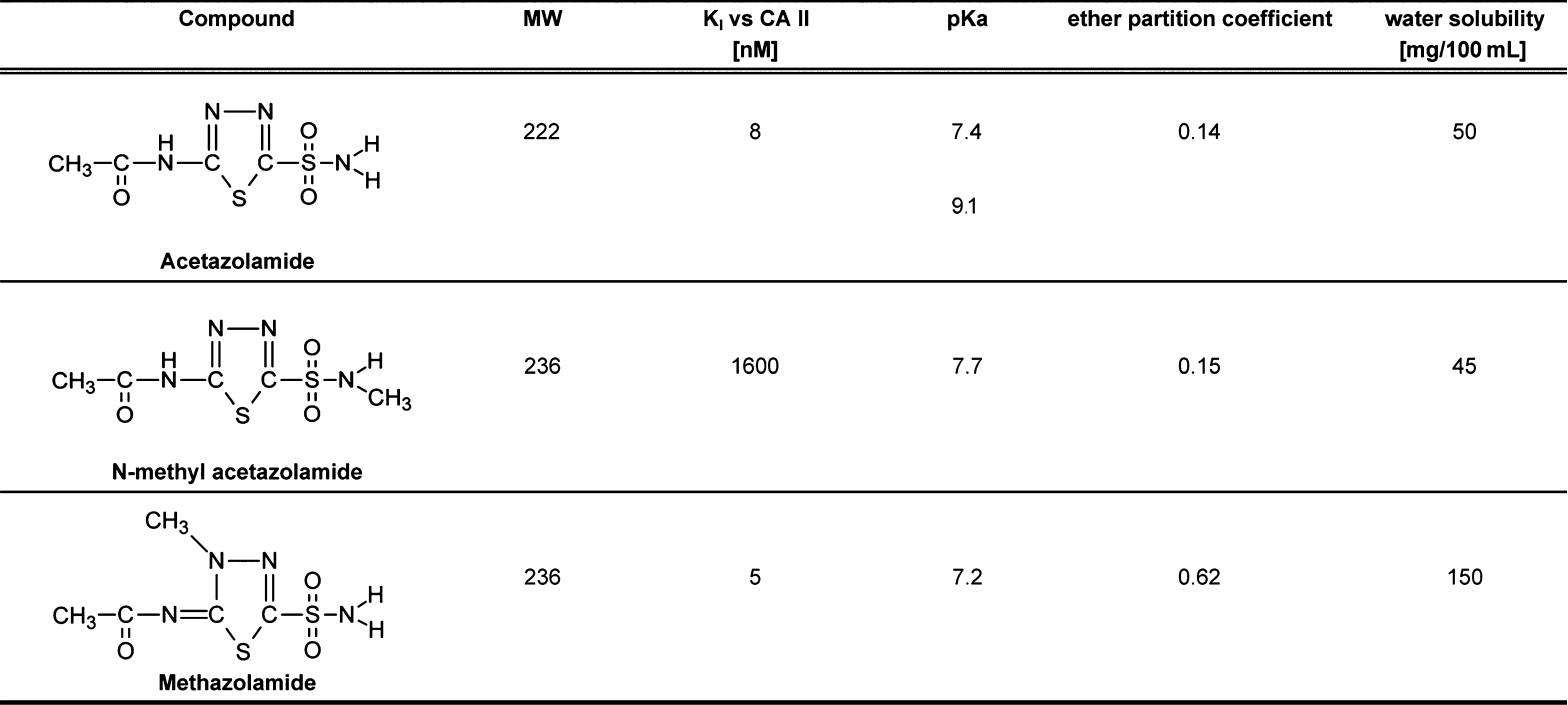
Structures, carbonic anhydrase (CA) inhibition constants, and chemical characteristics of n-methylacetazolamide (NMA), acetazolamide (AZ), and methazolamide (MZ).

The aim of the present study was to examine the effects of NMA on the control of breathing in the cat to directly and for the first time test whether AZ reduces the hypoxic ventilatory response (HVR) and decreases the hypercapnic ventilatory CO_2_ response by a mechanism unrelated to CA inhibition. We also wished to compare these effects with those previously reported for the nearly structurally related CA inhibitor, methazolamide. The chemical structures of the three compounds are given in Figure1. We hypothesized that NMA would reduce isocapnic HVR in a manner similar to AZ based on the results in the pulmonary vasculature. In contrast, we anticipated that it would not alter the ventilatory responses to CO_2_, which are reduced by both AZ and MZ and wherein CA-dependent rapid acid–base signaling processes would be expected to be more important.

## Methods

Experiments were performed in eight adult cats of either sex (mean bodyweight 3.6 ± 1.0 kg) after approval by the Ethical Committee for Animal Experiments of the University of Leiden. The animals were sedated with 10 mg kg^−1^ ketamine hydrochloride given intramuscularly. Anesthesia was induced with 2% sevoflurane in 30% O_2_ in N_2_. Both femoral arteries and the right femoral vein were cannulated, 20 mg kg^−1^ *α*-chloralose and 100 mg kg^−1^ urethane were slowly administered intravenously, and the volatile anesthetic was gradually withdrawn. An infusion of *α*-chloralose-urethane solution was then started at a rate 1.0–1.5 mg kg^−1^ per hour for *α*-chloralose and 5.0–7.5 mg kg^−1^ per hour for urethane to obtain a stable but light state of anesthesia sufficient to suppress pain withdrawal reflexes but light enough to preserve the corneal reflex. Under this anesthetic regimen, ventilatory parameters remain stable over several hours and are similar to those in animals awake (Gautier and Bonora 1979; Berkenbosch et al. 1997).

To measure inspiratory and expiratory flow, the trachea of the animals was cannulated at midcervical level and connected to a Fleisch Nr. 0 flow transducer (Fleisch, Lausanne, Switzerland), which was attached to a differential pressure transducer (Statham PM197, Los Angeles, CA). The flow transducer was connected to a T-piece of which one arm received a continuous fresh gas flow of 5 L min^−1^. Three computer-controlled mass flow controllers (Type AFC 60, High-Tec, Veenendaal, the Netherlands) generated desired inspiratory gas mixtures of oxygen, carbon dioxide, and nitrogen. The inspiratory and expiratory fractions of oxygen and carbon dioxide were measured with a Datex Multicap monitor (Datex-Engstrom, Helsinki, Finland), which was calibrated before each experiment with gases of known composition. The end-tidal pco_2_ and po_2_ were controlled independently by a computer by adjusting the inspiratory gas fractions. Body temperature was maintained between 37.0 and 37.6°C by means of a heating pad. After termination of the experiments, the animals were humanely killed with an overdose of pentobarbital. Arterial blood samples were taken from the right femoral artery for blood gas analysis (ABL 700, Radiometer Copenhagen, Denmark). Arterial blood pressure was measured using a Statham pressure transducer (P23ac, Los Angeles, CA). All signals were converted to digital values (sample frequency 100 Hz), processed by a computer, and stored breath-by-breath.

## Study design

We studied the effect of an intravenous dose of 20 mg kg^−1^ of *N*-methylacetazolamide (NMA; dissolved in distilled water to a concentration of 10 mg mL^−1^) on the acute ventilatory response to CO_2_ and on the isocapnic hypoxic response at arterial pco_2_ values that were kept the same before and after the drug infusion. Once a stable anesthetic state was established, we performed 3–4 dynamic end-tidal forcing (DEF) runs (see below) to assess the (dynamic) ventilatory response to CO_2_. Subsequently the steady response to isocapnic hypoxia was measured as described below.

### Ventilatory response to CO_2_

Using the DEF technique, we performed step changes in end-tidal pco_2_ before and after intravenous infusion of NMA. In order to avoid irregular breathing at pco_2_ values close to the apneic threshold, we clamped baseline end-tidal pco_2_ at a level approximately 3–4 mmHg higher than the apneic threshold during any given experimental condition (i.e., control and after each drug infusion). In both control and NMA conditions 2–4 DEF runs were performed at a normoxic background (P_E__T_o_2_ maintained at 13 kPa) and the dynamic ventilatory responses analyzed as described below. The P_E__T_co_2_ pattern during a DEF run was as follows. After a 10–15 min of steady-state ventilation at constant end-tidal pco_2_, P_E__T_co_2_ was increased by 1–1.5 kPa in a stepwise fashion and kept constant for 7 min. Thereafter, it was returned to its previous value and maintained for another 7 min.

### Analysis of dynamic ventilatory response to CO_2_

The steady-state relation between ventilation and Petco_2_ is linear down to apnea as described by.

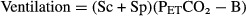


Where Sc and Sp are the ventilatory CO_2_ sensitivities of the central and peripheral chemoreflex loops and B represents the apneic threshold or extrapolated P_E__T_co_2_ at zero ventilation (De Goede et al. 1981, 1985). When applying rapid changes in end-tidal pco_2_ at constant end-tidal po_2_ it is possible to quantify the contributions of the peripheral and central chemoreflex loops to total ventilation. This is based on the difference in response times and dynamics of the two chemoreflex loops in response to a change in end-tidal pco_2_ (De Goede et al. 1985). The central chemoreflex loop displays a relatively large time delay (average response time in the cat is 8 sec) with slow dynamics (average time constant in the cat is 100 sec) while the response time of the peripheral chemoreflex loop is on average 4 sec with a time constant of about 10 sec (De Goede et al. 1985). To estimate Sc, Sp, and B, we fitted the ventilatory responses to a two-compartmental model using a least squares fitting routine as described previously (De Goede et al. 1985; Berkenbosch et al. 1992). In the fitting procedure, parameters were not restricted to values equal to or greater than zero. When negative values did occur we set the values to zero in the statistical analysis. To compare the means of the values obtained from the analysis of the DEF runs in the control situation with those obtained after NMA, a two-way analysis of variance was performed on individual data. The level of significance was set at *P *=* *0.05. Results are given as the average of the means per cat ± SD.

### Ventilatory response to isocapnic hypoxia

Near stepwise changes in end-tidal po_2_ were achieved by adjusting the O_2_ fraction of the inspired air. To avoid confounding influences of an O_2_–CO_2_ interaction in the carotid bodies, the end-tidal pco_2_ was kept constant by adjusting the inspired CO_2_ concentration to changing levels of ventilation (Berkenbosch et al. 1997; Teppema et al. 2006a). In this way, a new steady-state level of ventilation is established after about 6 min (Berkenbosch et al. 1997). Ventilation during the last 20 breaths of this period was averaged to yield steady-state ventilation at a given end-tidal po_2_. Blood samples were taken at the end of the steady-state periods to analyze arterial blood gases. Using a least square method, inspiratory ventilation *V*_*I*_ was fitted to Pao_2_ according to the exponential function (Berkenbosch et al. 1997; Teppema and Dahan 2010):

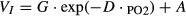
in which *G* is the overall hypoxic sensitivity (in L min^−1^), *D* is a shape parameter (kPa^−1)^ thought to be related to the shape of oxygen–hemoglobin saturation curve (Cunningham et al. 1986), and *A* is the minute ventilation during hyperoxia. These three parameters were estimated with the aid of an iteration method, but none of them was fixed. The number of data points were not necessarily equal in all treatments (normally ∼4 points in hyperoxia, ∼2 in normoxia, and ∼4 in hypoxia). However, the data points that were included were chosen such that within cats the Po_2_ range over which curve fitting was applied was the same in all treatments. To detect significant differences of parameters *G*, *A*, and *D* between treatments (control and 20 mg kg^−1^ NMA), we performed a two-way analysis of variance (ANOVA) (SPSS v11.0 for windows (SPSS Inc., Chicago, IL). *Ps *< 0.05 were considered significant. Data are presented as means ± SD.

## Results

In six cats, both hypercapnia and hypoxia were studied. In one cat only hypercapnia was studied and in another only hypoxia was studied. Altogether eight cats were studied.

### Ventilatory response to CO_2_

**Figure 2:**
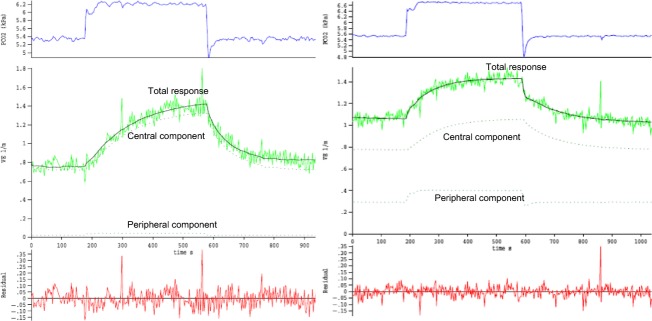
Example of two DEF curves in one animal before (left panel) and after (right panel) infusion of 20 mg kg^−1^ n-methylacetazolamide (NMA). For these two DEF runs, estimated parameter values for B (apneic threshold, i.e., the end-tidal pco_2_ with zero ventilation), Sc (= central CO_2_ sensitivity), and Sp (= peripheral CO_2_ sensitivity) in control and after infusion of 20 mg kg^−1^ NMA, respectively, were 4.39 and 2.64 kPa, 0.75 and 0.19 L min^−1^ kPa^−1^, and 0.09 and 0.09 L min^−1^ kPa^−1^, respectively. Upper traces: end-tidal pco_2_; middle traces: ventilatory response (continuous lines), composed of estimated central and peripheral contributions (stippled lines); lower traces: residual sum of errors.

In total, 27 control hypercapnic steps were analyzed and 22 after NMA administration. The mean results of the parameter estimations are presented in Table1. *N*-methylacetazolamide caused large decreases in the apneic threshold and both peripheral and central CO_2_ sensitivities, but did not alter delays and time constants (data not shown). An example in one animal is shown in Figure2. DEF runs after NMA infusion were performed at least 60 min after administration allowing sufficient time for wash-in based on our previous studies showing it takes 30–45 min after AZ infusion before respiratory effects fully develop (Wagenaar et al. 1996, 1998).

**Table 1:** Effects of *N*-methylacetazolamide (NMA) on the steady-state hypercapnic response in seven cats

	Control	NMA	*P*-value
Sc (L min^−1^ kPa^−1^)	0.593 ± 0.16	0.257 ± 0.04	0.002
Sp (L min^−1^ kPa^−1^)	0.116 ± 0.05	0.050 ± 0.04	0.002
Stot (L min^−1^ kPa^−1^)	0.700 ± 0.18	0.307 ± 0.07	0.001
Sp/Sc	0.260 ± 0.13	0.230 ± 0.18	0.678
B kPa	3.789 ± 0.92	1.341 ± 0.84	0.001

Sc, CO_2_ sensitivity of the central chemoreflex loop; Sp, CO_2_ sensitivity of the peripheral chemoreflex loop; B, apneic threshold, that is, P_E__T_co_2_ at zero ventilation.

### Ventilatory response to hypoxia

**Figure 3:**
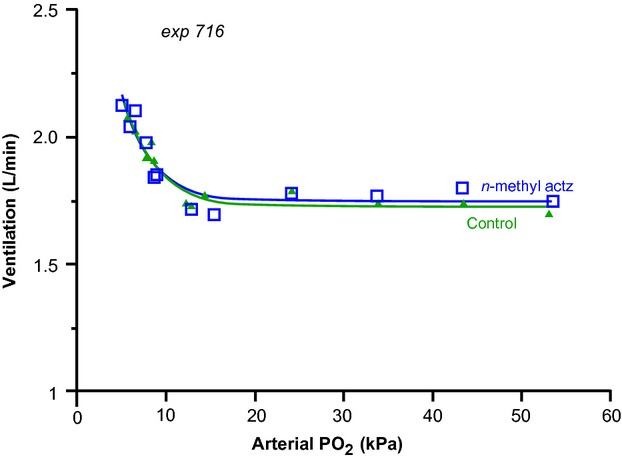
Isocapnic hypoxic response curves in one animal. Hypoxic response curves before and after n-methylacetazolamide (NMA) administration are not different. Parameter values for the shape factors D, gain G, and hyperoxic ventilation in control and after infusion of 20 mg kg^−1^ NMA in this animal were 0.29 and 0.31, 1.89 and 1.96 L min^−1^, and 1.73 and 1.75 L min^−1^, respectively. See text and legends to Table2 for the significance of these parameters.

Table2 summarizes the effect of NMA on the hypoxic response curve, measured at a constant Paco_2_ of about 5 kPa before and after NMA infusion. It shows that acid–base status before and after NMA were equal and unchanged, as expected for a drug without CA inhibiting activity. Hypoxic sensitivity *G* remained at a value of ± 2.6 to 2.7 L min^−1^. The shape parameter *D* remained at about 2.4–2.5 kPa^−1^, similar to that reported previously for morphine, AZ, and MZ (Berkenbosch et al. 1997; Teppema et al. 2006a). Parameter *A*, ventilation in hyperoxia, did not change. In Table2, note that NMA did not change the existing very small (<1 mmHg) and normal arterial-to end-tidal pco_2_ differences indicating the absence of erythrocytic CA inhibition, which generates very large and positive (>20 mmHg) gradients (Wagenaar et al. 1996; Kiwull-Schone et al. 2009). An example of isocapnic response curves before and after drug administration in one animal is shown in Figure3.

**Table 2:** Effects of *N*-methylacetazolamide (NMA, 20 mg kg^−1^) on the steady-state hypoxic response in seven cats

	Control	NMA	*P*
G (L min^−1^)	2.64 ± 0.87	2.69 ± 1.53	0.94
D (kPa^−1^)	0.24 ± 0.07	0.25 ± 0.10	0.81
A (L min^−1^)	1.14 ± 0.46	1.07 ± 0.48	0.38
Paco_2_ (kPa)	5.03 ± 0.39	5.18 ± 0.34	0.39
P(a-ET) co_2_ (kPa)	−0.62 ± 0.21	0.70 ± 0.31	0.64
Arterial pH	7.312 ± 0.018	7.304 ± 0.016	0.40
Base excess (mmol/L)	−6.5 ± 1.4	−6.8 ± 1.4	0.34

The hypoxic response is presented as an exponential function: *V*_1 _= *G*·exp(−*D*·po_2_ + A. G is the overall hypoxic sensitivity, that is, minute ventilation at very low PaO_2_. D is a shape parameter (kPa^−1^) thought to be related to the shape of oxygen–hemoglobin saturation curve (Cunningham et al. 1986), and A is the minute ventilation during hyperoxia. NMA has no effects on these parameters of hypoxic response curve. Note that NMA did not induce a rise in the P(a-ET) co_2_ gradient indicating the absence of erythrocytic carbonic anhydrase inhibition.

## Discussion

We have directly and for the first time studied whether some of the well-known effects of acetazolamide on the control of ventilation may be unrelated to its classical action as a carbonic anhydrase inhibitor. This has been indirectly suggested by differing results with various other CA inhibitors, such as methazolamide in particular, on certain aspects of ventilatory control, and also to differences in response to CA inhibitors on the hypoxic response of the pulmonary vasculature. In all of these experiments using potent CA inhibitors, however, it could never been fully ruled out that different responses might arise from differences in cellular uptake and distribution related to their physiochemical properties, rather than other targets of action. NMA, by virtue of its very similar structure and properties to AZ except a more than 200-fold weaker binding to CA, allowed us to examine this important question with greater certainty. In brief, using NMA, we find evidence that in regard to ventilatory responses to CO_2_, acetazolamide has actions unrelated to CA inhibition. With regard to the HVR, the finding that in contrast to AZ, NMA does not reduce it may suggest that in this case the effect of AZ results from CA inhibition. However, since even high-dose MZ does not reduce the HVR, this possibility is unlikely and indicates that in the carotid bodies the CA-independent action of AZ is not mimicked by NMA. It also illustrates the importance of comparing NMA’s effect with those of several (rather than only one) carbonic anhydrase inhibitors.

### Are the effects of NMA independent of CA inhibition?

*N*-methylacetazolamide is an acetazolamide analog in which one of the amine hydrogen atoms of the sulfonamide moiety (responsible for inhibition of CA by direct binding to the catalytically activated zinc ion in the active site) is replaced by a methyl group resulting in an approximately 200-fold decrease in binding affinity to carbonic anhydrase II, while its physical–chemical properties (Maren 1956; Duffel et al. 1986) are similar to that of AZ (Fig.1).

Given the very similar structure of NMA to AZ, it is possible that effects of NMA we and others report simply represent in vivo demethylation to AZ. Reported in vivo effects of NMA consistent with CA inhibition are a reduction in intraocular pressure in rabbits after topical administration (Duffel et al. 1986) and in the rat a very small renal AZ-like effect when very large concentrations or dosing were used (Maren 1956). These effects are, in fact, attributed to the small rate of in vivo demethylation of NMA to AZ – a 2% conversion in the rat after an oral dose of 100 mg kg^−1^ (Maren 1956; Duffel et al. 1986). In the dog, an intravenous dose of 11 mg/kg elicited no renal response and there was no AZ detected in the urine, despite active secretion of the drug by the kidney and concentration in the urine (Maren 1956). Furthermore, in the dog, no detectable AZ was found in blood 3 h following an intravenous loading dose of 10 mg kg^−1^ and a continuous infusion of 10 mg kg^−1^ per hour (detection threshold was 1 *μ*g mL^−1^; 25). One and 2 h after an intravenous dose of 68.5 mg kg^−1^ NMA in a cat, that is, threefold higher than we administered, the plasma AZ concentration was 2.5 and 2.7 *μ*g mL^−1^, respectively, accompanied by a small reduction in CSF flow rate equivalent to that with 0.5 mg kg^−1^ AZ (Maren 1956).

In the present study, cats received 20 mg kg^−1^ intravenously without any resulting increase in the arterial-to-end-tidal pco_2_ gradient (Table2). This finding suggests that the NMA did not induce inhibition of erythrocytic CA. Although we did not measure urine output and content to exclude a potential small effect of NMA on renal carbonic anhydrase activity, base excess remained unchanged highly suggestive of no increased urinary bicarbonate excretion, the signature of renal CA inhibition. Although we cannot exclude a very limited conversion of NMA to AZ; on the basis of literature data cited above, we consider it highly unlikely that the physiologic effects of NMA reported herein are mediated by AZ. Note that in a previous study we did not observe any respiratory effects of 0.5 mg kg^−1^ AZ (Wagenaar et al. 1996). Thus, taken in their entirety, these data give us strong confidence that we have observed unique, non-CA inhibiting effects of NMA on ventilatory responses.

### Decreases in the slope of the CO_2_ response curve and apneic threshold by AZ, MZ, and NMA are not related to CA inhibition

The effects of 20 mg kg^−1^ NMA on Sc, Sp, and the apneic threshold B reported herein are qualitatively similar to those obtained after 4 mg kg^−1^ AZ and 3 mg kg^−1^ MZ, doses that do not lead to physiologically significant inhibition of the intraerythrocytic enzyme (Wagenaar et al. 1996; Teppema et al. 2001; Bijl et al. 2006). The effects induced by AZ and MZ on CO_2_ responsiveness could be due to inhibition of an easily accessible plasma-facing carbonic anhydrase isoform at the luminal side of the blood–brain barrier altering the CO_2_ sensitivity of cerebral blood flow by slightly impairing CO_2_ exchange with capillary blood (Wagenaar et al. 1996; Teppema et al. 2001). This may explain the finding in the rabbit that 4 mg kg^−1^ intravenous AZ causes an approximately 25% rise in cerebral blood flow without changing Paco_2_ (Taki et al. 2001). However, the previously reported respiratory effects of AZ and MZ may occur independently from inhibition of CA, possibly by vasorelaxing effects on the cerebral vasculature thus altering the brain tissue–arterial pco_2_ relationship. The effects of NMA on the CO_2_ response curve, a shift to the left and decrease in slope that we report here are possibly mediated by a similar mechanism. Furthermore, in the same species, this dose weakens respiratory muscle function by affecting the transmission of CO_2_-induced increases in phrenic activity into differences in pleural pressure and tidal volume (Kiwull-Schone et al. 2001). This could provide another explanation for the reduced CO_2_ response. This effect on respiratory muscle function surprisingly is not shared by MZ (Kiwull-Schone et al. 2006, 2009) and suggests that in the rabbit AZ acts independently of CA inhibition to weaken respiratory muscle function as it appears also to do also in humans (Gonzales and Scheuermann 2013).

The striking similarities between the effects of NMA, AZ, and MZ in the cat (Table3) on the hypercapnic ventilatory response is highly suggestive of CA-independent effects of AZ and MZ, and we propose that similar to the lung described above and in other systemic vessels (Pickkers et al. 2001; Torring et al. 2009), NMA may have vasodilatory effects on cerebral vessels. While the mechanism for HPV inhibition remains unknown, in the systemic circulation the data point to a likely activation of Ca^2+^-activated potassium (BK) channels (Pickkers et al. 2001). A specific stimulating effect of AZ, but not MZ, on BK channels was shown in skeletal muscle cells from rat (Tricarico et al. 2000). Whether NMA shares all these vascular and muscular effects of AZ remains to be studied. NMA reduced the mean arterial blood pressure in the cats from 16.7 ± 4.3 to 14.9 ± 5.2 kPa, but this fall did not reach statistical significance (*P *=* *0.18). As yet, potential vasodilatory actions of MZ remain relatively unexplored. Note that in tissues other than muscle, MZ may be able to open BK channels depending on the membrane potential albeit with less potency than AZ (Tricarico et al. 2013) and that it has been reported to have physiological effects independently from CA inhibition on hepatic insulin sensitization (Konstantopoulos et al. 2012).

**Table 3:** Effects of acetazolamide (AZ), methazolamide (MZ), and *N*-methylacetazolamide (NMA) on pulmonary vascular and ventilatory responses

	AZ	MZ	NMA
iHVR			
Cat (i.v.)	Reduced^1^	Unaltered^12^	Unaltered^14^
Humans (i.v.)	Reduced^2^,^3^	Unknown	Unknown
Humans (oral)	Unchanged^3^,^4^	Unknown	Unknown
HCVR			
Cat (i.v.)	Reduced^5^	Reduced^13^	Reduced^14^
Rabbit (i.v.)	Reduced^6^	Unaltered^13^	Unknown
Humans (i.v.)	Unaltered/reduced^3^	Unknown	Unknown
Humans (oral)	Unaltered^7^/increased^3^	Unknown	Unknown
Respiratory muscles			
Rabbit (i.v.)	Impaired^6^	Unaltered^14^	Unknown
Humans (oral)	Impaired^15^	Unknown	Unknown
HPV			
Humans (oral)	Reduced^8^,^9^	Unknown	Unknown
Dogs (i.v.)	Reduced^10^,^11^	Reduced^10^	Reduced^10^

Comparison of the effects of acetazolamide (AZ), methazolamide (MZ), and *N*-methylacetazolamide (NMA) on pulmonary vascular and ventilatory responses. In all cases, doses lower than those needed to induce effective red cell CA inhibition were administered. iHVR, isocapnic hypoxic ventilatory response; HCVR, hypercapnic ventilatory response; HPV, hypoxic pulmonary vasoconstriction.

1 Teppema et al. (2001); Teppema and Dahan (2004)

2 Swenson and Hughes (1993); Teppema et al. 2006b)

3 Swenson and Hughes (1993)

4 Teppema et al. (2010) – note that in this study hypoxic sensitivity was defined as the ratio delta log Pao_2_ over delta ventilation since the HbO_2_ saturation curve undergoes a Bohr shift due to the acidosis induced by chronic AZ

5 Wagenaar et al. (1996, 1998)

6 Kiwull-Schone et al. (2001)

7 Teppema and Dahan (1999)

8 Teppema et al. (2007)

9 Ke et al. (2013)

10 Pickerodt et al. (2014)

11 Hohne et al. (2004)

12 Teppema et al. (2006a)

13 Bijl et al. 2006) – note that only central CO_2_ sensitivity was reduced)

14 Kiwull-Schone et al. (2009), this study

15 Gonzales and Scheuermann (2013).

An effect of NMA on respiratory muscles is unknown and remains to be studied, although its failure to reduce the hypoxic response (discussed below) does not support a decrease in muscle efficiency. Finally, we cannot exclude a direct action of NMA on the CNS since we have no information on its ability to cross the blood–brain barrier. Note that the agent did not change the ratio of Sp over Sc, which might suggest an action on the respiratory integrating centers in the brain. However, NMA’s lipid and water solubility are very similar to AZ (Fig.1), and it is unlikely that it readily enters the brain.

### NMA does not influence the ventilatory response to hypoxia

The finding that in contrast to AZ, NMA did not reduce the hypoxic ventilatory response may suggest that the previously reported inhibitory effect of AZ (see Table3) is due to CA inhibition. However, the situation is not entirely clear because even a higher dose of the much more permeable inhibitor MZ does not alter the HVR, while subsequent administration of AZ after MZ, thus in the presence of an already completely inhibited enzyme, reduces it (Teppema et al. 2006a). In fact, different effects of AZ and MZ have also been demonstrated at the level of the carotid sinus nerve (CSN). In the in vitro cat carotid body, MZ abolishes the initial overshoot of a step hypoxia-induced increase in discharge frequency but the steady-state change is unaffected (Iturriaga et al. 1993). AZ, however, reduces the steady-state CSN response in vivo (Lahiri et al. 1976). Comparison of the effects of NMA, AZ, and MZ is complicated by the fact that a great fraction of MZ is metabolized to unique metabolites not observed with AZ and NMA, despite very similar structures (Kishida et al. 2011). In the cat, 70% is metabolized and 30% excreted unchanged with the urine (Vogh 1980).

The unexpected absence of any effect of NMA on the HVR, while the hypoxia-induced rise in (Ca^2+^_i_) in PASMCs and HPV are reduced (Hohne et al. 2004; Shimoda et al. 2007), may be the consequence of different cellular mechanisms of oxygen sensing in type I carotid body cells and PASMCs. Another reason could be a low accessibility of NMA to carotid body tissue compared to AZ after 4 mg kg^−1^. However, unless NMA has a much higher plasma binding than AZ, this seems unlikely because NMA was given in the much higher dose of 20 mg kg^−1^ and its pK_a_ of 7.7 is somewhat higher than the 7.4 of AZ (Maren 1956) with about equal lipid solubility (see Fig.1).

### Summary and prospects

With regard to the hypercapnic ventilatory response, the finding that NMA, AZ, and MZ have very similar effects suggests to us that AZ and MZ act independently of carbonic anhydrase inhibition to reduce this response. This may be of clinical relevance since AZ is used in a variety of disease states such as sleep apnea, heart failure, and acute mountain sickness whereby its exact action mechanism(s) remain unclear (Swenson et al. 1991; Swenson and Hughes 1993; Javaheri 2006; Edwards et al. 2012; Apostolo et al. 2014). Note that AZ has a broad spectrum of actions that could contribute to its physiologic effects (Teppema 2014). Avoiding the side effects of CA inhibition, use of NMA in humans could be an interesting step in elucidating the underlying mechanisms of the clinical effects of AZ.

In conclusion, what we have learned by comparing the effects of AZ, MZ, and NMA on HVR are the following. First, HVR is not dependent on CA activity since the highly lipophilic inhibitor MZ causes no change. Second, because MZ and NMA on one hand and AZ on the other hand have different effects on the carotid bodies, a challenge for future studies is to look for common effects of MZ and NMA at the cellular level, for example, in PASMCs that are not shared by AZ. With regard to the ventilatory responses to CO_2_, it remains critical that uncertainties around how these drugs affect cerebrovascular tone and cerebral blood flow and thus local pH and CO_2_ concentrations in the brain need to be better understood. Third, as emphasized by Maren more than 35 years ago (Maren 1977), more clinical studies with methazolamide are warranted (see also Table3). The data from the animal studies that we performed may suggest that MZ may be a reasonable and perhaps better alternative for AZ in clinical applications. Finally, given the sometimes surprising and distinctively differing results of three such structurally similar molecules, the heavy and sole reliance upon AZ as a definitive pharmacological probe for CA function in a number of physiological systems should be reconsidered (Maren 1977).

## Conflict of interest

None declared.
